# Carbopol-Incorporated Thermoreversible Gel for Intranasal Drug Delivery

**DOI:** 10.3390/molecules20034124

**Published:** 2015-03-04

**Authors:** Prabagar Balakrishnan, Eun-Kyoung Park, Chung-Kil Song, Hyun-Jeong Ko, Tae-Wook Hahn, Ki-Won Song, Hyun-Jong Cho

**Affiliations:** 1 College of Pharmacy, Hanyang University, Ansan 426-791, Korea; 2 Department of Organic Material Science & Engineering, Pusan National University, Busan 609-735, Korea; E-Mails: ek31004@hanmail.net (E.-K.P.); kwsong@pusan.ac.kr (K.-W.S.); 3 College of Pharmacy and Research Institute of Pharmaceutical Sciences, Seoul National University, Seoul 151-742, Korea; E-Mail: saintck@naver.com; 4 College of Pharmacy, Kangwon National University, Chuncheon 200-701, Korea; E-Mail: hjko@kangwon.ac.kr; 5 College of Veterinary Medicine and Institute of Veterinary Science, Kangwon National University, Chuncheon 200-701, Korea; E-Mail: twhahn@kangwon.ac.kr

**Keywords:** fexofenadine hydrochloride, nasal delivery, thermoreversibility, poloxamer, carbopol, bioavailability

## Abstract

The present study describes the preparation and evaluation of a poloxamer 407 (P407)-based thermoreversible gel using Carbopol 934P (C934P) as a mucoadhesive polymer and hydroxypropyl-β-cyclodextrin (HP-β-CD) for enhancing the aqueous solubility and intranasal absorption of fexofenadine hydrochloride (FXD HCl). The prepared gels were characterized by gelation temperature, viscoelasticity, and drug release profile. Thermoreversibility of P407/C934P gel was demonstrated by rheological studies. The incorporation of carbopol into P407 gel also reduced the amounts of drug released from the gel formulations (*p* < 0.05). *In vivo* pharmacokinetic results of the prepared gel formulations in rabbits (at 0.5 mg/kg dose) showed that the relative bioavailability of drug from P407/C934P gel was 11.3 and 2.7-fold higher than those of drug solution and P407 gel group, respectively. These findings suggested that developed thermoreversible gels could be used as promising dosage forms to improve intranasal drug absorption.

## 1. Introduction

Intranasal (IN) delivery has often considered as an alternative to the intravenous route for diverse therapeutic compounds [1]. This is due to the large surface area of the nasal mucosa that affords a rapid onset of therapeutic effect, potential for direct delivery to the central nervous system via the olfactory pathway, no first-pass effect, and non-invasiveness [2,3]. Though IN delivery has limits for macromolecular drugs (*i.e.*, nucleic acids, peptides, and proteins), cell tight junctions can be temporarily opened and the paracellular route can be used for the transport of those drugs [4]. IN administration is a painless route and it can be used in emergency situations by the patient or a physician. However, the small administration volume due to the restricted volume of the nasal cavity can be a disadvantage for the development of IN formulations. Consequently, only low dosage or highly soluble drugs can be candidates for IN formulations. Also, mucociliary clearance system can remove the formulations rapidly (~21 min) from the nasal cavity and nasal irritation of drug or formulation components has to be taken into account while developing IN formulations [5,6].

Fexofenadine hydrochloride (FXD HCl), a non-anticholinergic H1-receptor antagonist devoid of sedative effects, is a long acting antihistamine widely used for the treatment of several allergic rhinitis symptoms [7]. FXD is classified into biopharmaceutical classification system (BCS) class III with a low human intestinal permeability [8,9]. Hepatic metabolism of FXD is less than 5% and its transport can be influenced by P-glycoprotein (P-gp) and organic anion transporting polypeptide (OATP) [8,10,11,12]. Oral bioavailability of FXD is known to be variable and low; 2%–4% in rodents, 6.6% in monkeys, and 30%–35% in humans [13,14,15,16]. Though a FXD tablet (Allegra^®^) for oral administration is available in the market, new formulations are required to enhance the systemic exposure of FXD as well as maintaining adequate local drug concentrations at the site of action.

Aqueous solutions do not serve well as IN formulations because they provide low bioavailability due to fast drug clearance by ciliary movements in the nasal cavity [2]. Therefore, mucoadhesive polymers could serve well to increase the residence time of the IN formulation, consequently increasing its bioavailability [17,18,19]. However, if the IN formulation is a gel at room temperature, it will be difficult to administer, hence a nasal thermoreversible gel could better serve IN applications as it remains liquid at room temperature and transforms into the gel at body temperature [5].

Poloxamer 407 (P407), a triblock copolymer consisting of a central hydrophobic block of polypropylene glycol flanked by two hydrophilic blocks of polyethylene glycol (PEO–PPO–PEO), can be used for development of *in situ* gelling systems. P407 aqueous solutions within certain concentration range show thermoreversibility, which is of great interest for optimizing drug formulations. The thermogelling phenomenon is completely reversible and can be characterized by the transition between sol and gel. Above this temperature, the solution becomes a semi-solid form while it remains as a liquid state below that temperature. The thermogelation results from interactions between different segments of the copolymer [20]. As the temperature increases, P407 copolymer molecules aggregate into micelles [21]. P407 has been reported to neither cause skin irritation nor sensitivity, and hence, it has good tolerability and usefulness in topical, rectal, and ocular formulations [21]. Carbopol is a homo- and copolymer of acrylic acid crosslinked with a polyalkenyl polyether and has been used in suspending agents, stabilizers, and thickeners. It is interesting to note that carbopol has been used to provide mucoadhesiveness for IN formulations [22,23].

This study investigated a thermoreversible gel formulation for IN delivery of FXD HCl, using P407 as a thermoreversible polymer, carbopol (C934P) as a mucoadhesive polymer and gelling agent, and hydroxypropyl-β-cyclodextrin (HP-β-CD) as a solubilizing agent and permeation enhancer. The thermoreversible nasal gels were characterized by gelation temperature, viscoelasticity and *in vitro* release studies. The developed thermoreversible gels were also assessed by an *in vivo* pharmacokinetic study in rabbits.

## 2. Results and Discussion

### 2.1. Gelation Temperature (T_sol-gel_)

Mucociliary clearance is one of the important limitations in nasal drug delivery; hence, thermoreversible gels could be a promising formulation option to overcome this problem. In this study, we formulated poloxamer and carbopol-based thermoreversible gel formulations for enhancing the bioavailability of the drug (Table 1). The suitable temperature range for nasal application has been reported in the range of 25 to 37 °C [5]. Therefore, the gelling temperature of the product should be above 25 °C to avoid difficulties in manufacturing, handling, and administering the formulation [24]. The nasal cavity temperature is around 34 °C [25], hence, we aimed at preparing P407-based thermoreversible gel that might be in a gel state in the range of 25 to 34 °C and a liquid state below 25 °C. It has been reported that the sol to gel transition temperature decreases when the P407 concentration increases [21]. In this investigation, P407 was fixed at 17% concentration for an appropriate gel transition temperature in the nasal cavity and the influence of carbopol incorporation into the P407 gel on the transition temperature was also evaluated. The sol-gel transition temperature of the developed formulation is presented in Table 2. The gelation temperatures of formulations were around 29–30 °C. The gelation temperature of carbopol-included poloxamer gel was slightly lower than that of reported P407-based gel formulation (29.9 ± 0.1 °C) [5], which is in good accordance with the previous report [17]. Thus, prepared formulations will be a liquid state at room temperature and transform into the gel state after instillation into the nostril. The thermoreversibility of P407/C934P gel may contribute to the increase of contact time in the nasal cavity.

**Table 1:** Compositions of prepared drug-loaded thermoreversible gel.

Formulation	HP-ß-CD Concentration (%, w/v)	P407 Concentration (%, w/v)	Carbopol Type (0.1%, w/v)
P407 gel	10	17	-
P407/C934P gel	10	17	C934P

FXD concentration was 1% (w/v) in all formulations.

**Table 2:** Gelation temperature of drug-loaded thermoreversible gel formulations with tilting method.

Formulation	Gelation Temperature ± S.D. (°C)
P407 gel	29.9 ± 0.1 ^a^
P407/C934P gel	29.7 ± 0.1

Each data represents the mean ± SD (n = 3). ^a^ This value was quoted from our previous study [5].

### 2.2. Viscoelastic Properties of the Thermoreversible Gels

**Figure 1:**
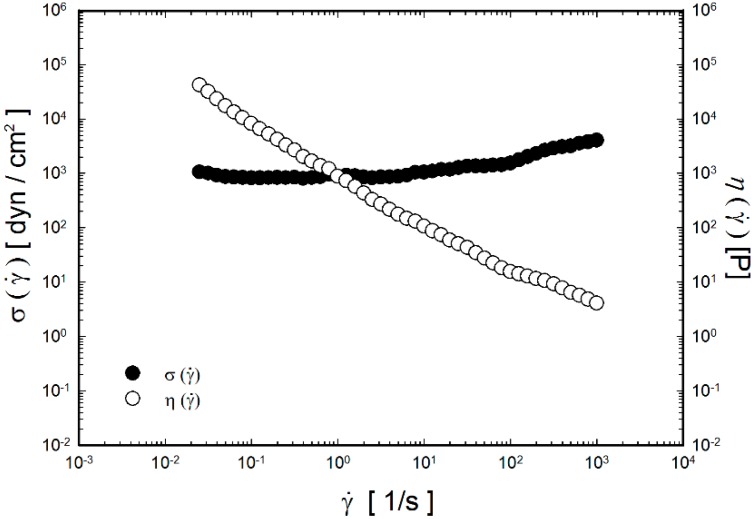
Shear stress (σ) and steady shear viscosity (η) as a function of shear rate for P407/C934P gel at 35 °C.

**Figure 2:**
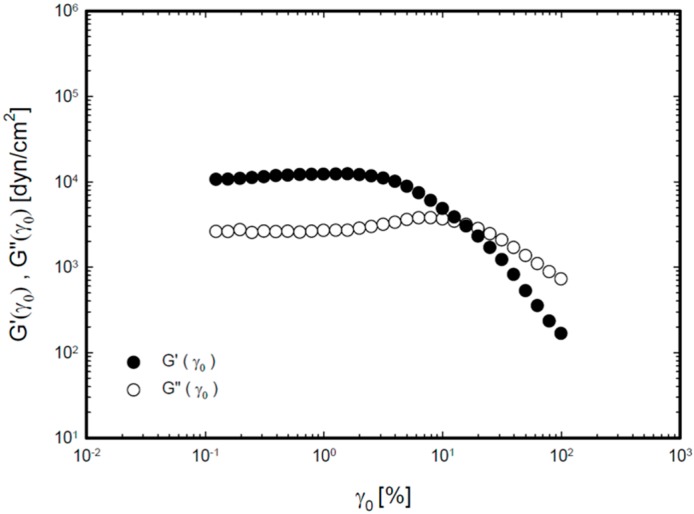
Storage modulus (G') and loss modulus (G'') as a function of strain amplitude (γ_0_, %) for P407/C934P gel at 35 °C.

**Figure 3:**
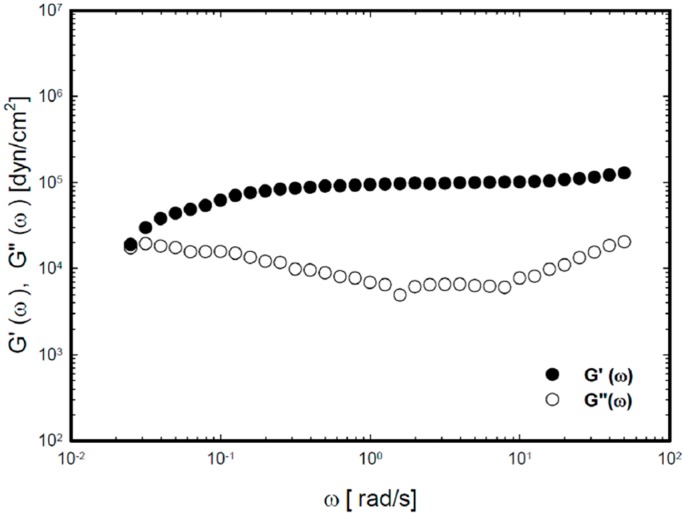
Storage modulus modulus (G') and loss modulus (G'') as a function of angular frequency (ω, rad/s) for P407/C934P gel at 35 °C.

The rheological characteristics of the P407/C934P thermoreversible gel containing FXD HCl were investigated as shown in Figure 1, Figure 2 and Figure 3. Those of P407 gel were already presented in our previous report [5], thus they are not included in this study. The viscosity of P407/C934P gel according to shear rate at 35 °C is shown in Figure 1. In the tested range of shear rate (0.025–1000 s^−1^), viscosity is decreased as the shear rate is increased. Shear-thinning behavior was observed in the profile of the developed thermoreversible gel (P407/C934P gel) and the fluidity was increased as the shear rate was increased. This shear-thinning behavior at 35 °C indicated the formation of gel above sol-gel transition temperature, as presented in Table 2.

Figure 2 demonstrates the storage (elastic) modulus (G') and loss (viscous) modulus (G'') of P407/C934P gel according to strain amplitude at a fixed angular frequency. The elastic modulus exhibited linear behavior up to 1% strain amplitude and a decreasing pattern above that value. The viscous modulus had a linear profile at <10% strain amplitude and decreasing behavior above that value.

The viscoelastic characteristics of P407/C934P gel according to angular frequency are shown in Figure 3. Because the elastic modulus and viscous modulus were commonly constant in the 0.1%–1% range of strain amplitude, the strain amplitude was fixed as 0.2% in the following frequency-sweep test. The elastic modulus and viscous modulus were measured according to the angular frequency range (0.025–50 rad/s). Over all angular frequency range, the elastic modulus was higher than the viscous modulus. Little fluctuation in elastic modulus implies the gel formation at 35 °C. All of these viscoelastic properties support the thermoreversibility of P407/C934P gel formulation in the nasal cavity.

### 2.3. In Vitro Drug Release Study

**Figure 4:**
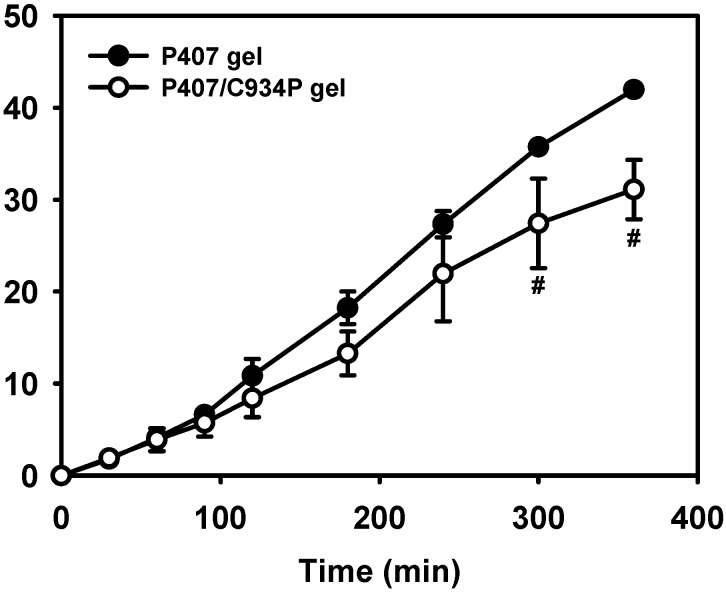
Drug release profiles from thermoreversible gel formulations. ^#^ *p* < 0.05, compared to P407 gel group. Each point represents the mean ± S.D. (n = 3).

The drug release profiles from gel formulations are shown in Figure 4. During the development of thermoreversible gel formulations, drug was complexed with HP-β-CD to increase its aqueous solubility. Without the addition of HP-β-CD, a determined drug concentration (10 mg/mL) in the gel cannot be accomplished. The released amounts and release rate can be reduced as the polymer is added to the gel formulations [26]. It seems that the addition of carbopol into the P407 gel structure may influence the aqueous channels of poloxamer micelles for drug diffusion. It can explain the retardation of drug release from P407/C934P gel compared to P407 gel after incubating for 6 h (*p* < 0.05). Though carbopol reduced the amounts of drug released from the gel formulation, it is expected that it can contribute to improve nasal drug absorption via mucoadhesiveness and permeation enhancement as reported [26].

### 2.4. In Vivo Pharmacokinetic Study

**Figure 5:**
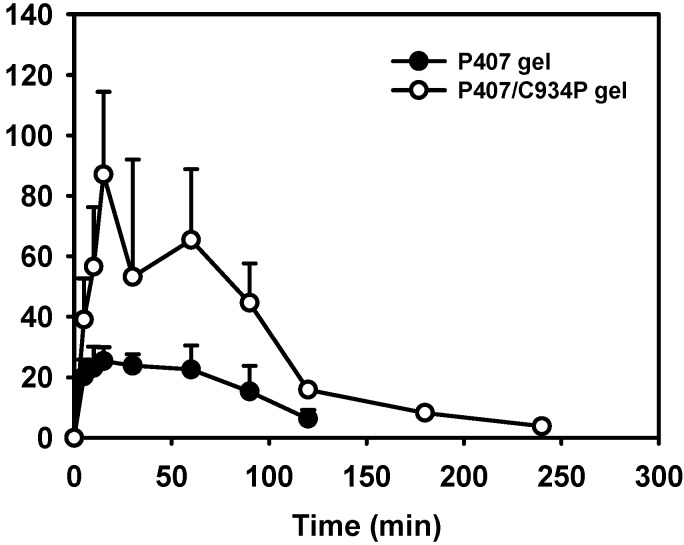
Mean drug concentration in plasma-time profiles after intranasal administration of thermoreversible gel formulations in rabbits. Dose was 0.5 mg/kg. Each point represents the mean ± S.D. (n ≥ 3).

*In vivo* absorption of FXD HCl following nasal administration of thermoreversible gels was compared to IN administration of drug aqueous solution (Table 3 and Figure 5) [5].

**Table 3:** Pharmacokinetic parameters of drug from various formulations administered by intravenous and nasal routes in rabbits.

Administration Route (formulation)	C_max_ (ng/mL)	AUC/D (kg·min/mL) × 10^−6^	Relative Bioavailability (%)
IN (solution) ^a^	11.7 ± 1.3 *	1264.4 ± 604.7 *	100
IN (P407 gel)	29.5 ± 3.1 *	5265.1 ± 1384.0 *	416
IN (P407/C934P gel)	94.7 ± 14.3 *	14323.6 ± 3773.6 *	1133

Each data represents the mean ± S.D. (n ≥ 3). ^a^ Pharmacokinetic parameters of IN (solution) group were taken from our previous study [5]. * *p* < 0.05, all values are different from each other.

In our previous report [5], the drug permeability of the P407 gel-treated group was significantly higher than that of drug aqueous solution in human nasal epithelial (HNE) cell monolayer system. It may be due to the absorption enhancing effect of HP-β-CD contained in P407 gel formulation. In this study, the pharmacokinetic properties of thermoreversible gel formulations were compared to the reported values of drug aqueous solution administered intranasally in rabbits [5]. Rabbits remained conscious throughout the experiment, thus functional mucociliary transport could work during the whole procedure.

The corresponding relative bioavailability and pharmacokinetic parameters are shown in Table 3. The relative bioavailability and C_max_ values of drug were in the following order: drug solution < P407 gel < P407/C934P gel (*p* < 0.05). Given that the absolute bioavailability of FXD after IN administration was 1.73% in our previous study [5], the absolute bioavailability of drug from P407/C934P gel is expected to be 19.60%. In the case of drug solution, the low absorption may be due to the faster clearance by mucociliary movement in the nasal epithelium. The thermoreversibility of P407 gel exhibited a significant enhancement of drug absorption after IN administration (*p* < 0.05). Enhanced drug absorption from P407/C934P gel may be explained by the combined effects of carbopol and HP-β-CD. In a biological environment, carbopol can prolong the contact time of thermoreversible gel formulations with the mucosal surface [26]. It was also known that carbopol can modulate cellular tight junctions thus enhancing paracellular drug transport [26]. They can contribute to elevate the systemic exposure of drug after IN application of carbopol-included gel, compared to P407 gel (*p* < 0.05). HP-β-CD included in the gel also contributed to improve the aqueous solubility and permeability of drug in this study. The enhanced drug release was a result of the incorporated HP-β-CD dissolving upon contact with water, increasing the porosity of the matrix and also allowing the removal of the drug via its inclusion within the HP-β-CD cavity [27,28]. The permeation enhancing effect of HP-β-CD, via several mechanisms, across mucosal membrane was also reported [5,29].

P407 and chitosan-based thermoreversible gel was already developed for intranasal delivery of FXD HCl in our previous study [5]. Elevation of systemic exposure of drug after IN administration of thermoreversible gel formulations was shown in that study. As in P407/chitosan gel, the addition of carbopol (0.1%) to P407 gel produced a comparable drug bioavailability. Considering the biocompatibility of carbopol [30,31], the prepared P407/C934P thermoreversible gel can be a promising nasal delivery vehicle for FXD HCl.

## 3. Experimental Section

### 3.1. Materials

FXD HCl was provided by Handok Pharmaceuticals Corp. (Seoul, Korea). Propranolol hydrochloride and HP-β-CD were obtained from Sigma-Aldrich Co. (St. Louis, MO, USA). P407 (Lutrol F-127) was a kind gift from BASF (Ludwigshafen, Germany). C934P was purchased from BF Goodrich Co. (Cleveland, OH, USA). BEGM BulletKit was obtained from Cambrex Bio Science Inc. (Walkersville, MD, USA), and other cell culture reagents were acquired from Invitrogen Co. (Grand Island, NY, USA). Transwell (0.4 µm pore size, 12 mm diameter, polyester) was purchased from Costar Co. (Cambridge, MA, USA). All other chemicals and reagents were of analytical grade and purchased from commercial sources.

### 3.2. Preparation of P407-Based Thermoreversible Gel

Thermoreversible P407 gels were prepared by the cold method as reported [5,32]. As shown in Table 1, P407 (17%, w/v) was dissolved in deionized distilled water (DDW) including HP-β-CD (10%, w/v) and drug (10 mg/mL) at room temperature, and it was stored at 4 °C for complete solubilization of P407. Carbopol (0.1%, w/v) solubilized in DDW was then slowly added to prepared solution with continuous agitation at 4 °C. Then, it was kept at 4 °C for 24 h before their use.

### 3.3. Measurement of Gelation Temperature

Gelation was evaluated by reported method [33]. Aliquots (1 mL) of prepared formulation were sealed and transferred to test tubes in the water bath (CW-05G, Jeio Tech Co. Ltd., Seoul, Korea) at 4 °C. Temperature was increased 2 °C per step until 25 °C and then with 1 °C increment (or 0.2 °C in the region of the gel-sol transition temperature). Gelation is the temperature that the meniscus would no longer move upon tilting through 90°. The method was very reproducible, giving coefficients of variation of less than 2% (n = 4).

### 3.4. Rheological Study

Rheological characteristics of developed P407/C934P gel were investigated by reported method with slight modification [5]. Rheological properties of drug-loaded P407/C934P gel was investigated using a strain-controlled rheometer (Advanced Rheometric Expansion System, ARES; Rheometric Scientific, Piscataway, NJ, USA) equipped with a cone-plate fixture having 12.5 mm radius and 0.04 rad cone angle of as well as a parallel-plate fixture having 12.5 mm radius and 0.5 mm gap size at 35 °C. The steady shear flow properties were measured at over a wide range of shear rate from 0.025 to 1000 s^−1^ with a logarithmically increasing scale. In this shear rate-sweep study, before the prepared sample was loaded, the lower and upper plates were covered with sandpaper in order to remove a wall slippage between the test material and the plates. For investigating the nonlinear viscoelastic profiles in large amplitude oscillatory shear flow fields, dynamic strain-sweep test was performed at 0.1%–100% strain amplitude and 1 rad/s angular frequency. In the frequency-sweep test, it was done at 35 °C isothermal condition over an angular frequency range from 0.025 to 50 rad/s, with a logarithmically increasing scale at fixed strain amplitude of 0.2%. In all measurements, a fresh sample aliquot was used and rested for 10 min after loading to allow material relaxation and temperature equilibration. All measurements were done in triplicate and highly reproducible data (coefficients of variation < 5%) were acquired.

### 3.5. In Vitro Release Study

A diffusion system was employed for *in vitro* release test. Dialysis membrane with 3.5 kDa molecular weight cut-off (MWCO, Spectrum Laboratories, Inc., Piscataway, NJ, USA) was used. The aliquots of formulation (1 mL) were loaded into the dialysis bag and placed in a glass beaker containing 100 mL phosphate buffered saline (PBS, pH 6.4), maintained at 37 °C in a water bath prior to release test. The release media was then stirred with 100 rpm speed. The samples (1 mL) were withdrawn at pre-determined times (5, 15, 30, 60, 90, 120, 180, and 360 min) and the equivalent volume of fresh media was replaced.

The released amount of drug in the media was quantitatively determined by high performance liquid chromatography (HPLC) system (Waters Co., Milford, MA, USA) equipped with a reverse phase C-18 column (Lichrospher^®^ 100, RP-18, 125 mm × 4 mm, 5 µm, Merck, Darmstadt, Germany), a pump (Waters 515 pump), an automatic injector (Waters 717plus autosampler), and UV detector (Waters 2487) [5]. The mobile phase was consisted of phosphate buffer (pH 3.0) and acetonitrile (70:30, v/v) and the eluent was monitored at 220 nm wavelength with 1.0 mL/min flow rate. The injection volume was 20 µL. The inter- and intra-day variance of this HPLC method was within the acceptable range.

### 3.6. In Vivo Pharmacokinetic Study

*In vivo* pharmacokinetic study was done in New Zealand white rabbits (2.7 ± 0.2 kg body weight). They were reared in a light-controlled room with a temperature of 22 ± 2 °C and a relative humidity of 55% ± 5% (Animal Center for Pharmaceutical Research, College of Pharmacy, Seoul National University, Seoul, Korea). The experimental protocols regarding animal study were approved by the Animal Care and Use Committee of the College of Pharmacy, Seoul National University (No. SNU-130722-1-1).

Prior to animal study, rabbits were fasted overnight (12 h) with free access to water. As reported [5], for IN administration group, drug solution and drug-loaded thermoreversible gel formulations (P407 gel and P407/C934P gel) (135 ± 10 µL) were administered into both nostrils of each rabbit at a 0.5 mg/kg dose. Blood samples (~1 mL) were collected from the ear vein at predetermined time intervals. Plasma samples were separated by centrifuging the blood samples at 7000 *g* for 5 min, and 500 µL aliquots were transferred to new tubes and stored at −20 °C before analysis. The pharmacokinetic parameters were calculated using the WinNonlin^®^ software (Version 3.1, Pharsight Co., Mountain View, CA, USA). The relative bioavailability was obtained by the following equation:

(1) $$Bioavailability\left(\%\right)=\frac{AUC_{formulation}/D_{formulation}}{AUC_{solution}/D_{solution}}$$

FXD HCl concentration in the plasma samples was determined by liquid chromatography-tandem mass spectrometry (LC-MS/MS) according to the reported method [5,34]. In brief, 50 µL of internal standard (propranolol, 1 µg/mL) was added to plasma samples (500 µL) and mixed with acetonitrile (1 mL). That mixture was vortexed for 10 min and centrifuged at 13,200 rpm for 5 min. The supernatant was transferred to new tubes and the solvent was removed by heating at 60 °C under the nitrogen gas stream. The residue was reconstituted with aliquot of mobile phase (100 µL). Aliquots (10 µL) were injected onto the LC-MS/MS system equipped with LC modules (Waters e2695) and LCQ advantage ion-trap mass spectrometer (Thermo Fisher Scientific Inc., Waltham, MA, USA). Gemini 3 μm C18 column (150 mm × 2 mm, 3 µm, Phenomenex, Torrance, CA, USA) was used at a flow rate of 0.2 mL/min with a mobile phase consisted of acetonitrile and water (30:70, v/v) including 0.1% formic acid. Electrospray ionization was performed in the positive mode with 380 °C probe temperature and *m/z* values of precursor to product ion at multiple reaction monitoring (MRM) mode were 502.3 to 466.2 for FXD and 260.2 to 183.1 for propranolol, respectively. Other operational parameters of MS/MS detector were optimized using built-in tuning system.

### 3.7. Data Analysis

All the experiments in the study were repeated at least three times and the data were expressed as the mean ± standard deviation (S.D.). Statistical analysis of data was performed using two-tailed *t*-test or analysis of variance (ANOVA). A *p*-value less than 0.05 was considered as significant.

## 4. Conclusions

A P407-based thermoreversible gel containing C934P (for mucoadhesiveness), and HP-β-CD (for improving solubilization and permeation of drug) was prepared for IN delivery of FXD HCl. The thermoreversibility of P407/C934P gel was verified by rheological studies. According to the results of pharmacokinetic study in rabbits, the relative bioavailability of drug from P407/C934P gel was 11.3 and 2.7-fold higher than those of drug solution and P407 gel group, respectively. All of these results revealed that developed thermoreversible gel can be a promising IN dosage form.
